# A Comprehensive Method with Verification for Characterizing the Visco-Hyperelastic Material Model of Polyurethane Foam of Passenger Car Seats

**DOI:** 10.3390/ma18153526

**Published:** 2025-07-28

**Authors:** Jianjiao Deng, Zunming Wang, Yi Qiu, Xu Zheng, Zuofeng Pan, Jingbao Zhao, Yuting Ma, Yabao Li, Chi Liu

**Affiliations:** 1 State Key Laboratory of Advanced Vehicle Integration and Control, China FAW Group Co., Ltd., Changchun 130011, China; dengjianjiao@faw.com.cn (J.D.); panzuofeng@faw.com.cn (Z.P.); zhaojingbao@faw.com.cn (J.Z.); mayuting1@faw.com.cn (Y.M.); liyabao@faw.com.cn (Y.L.); 2 Department of Energy Engineering, Zhejiang University, Hangzhou 310027, China; zm.wang@zju.edu.cn (Z.W.); chi.liu@zju.edu.cn (C.L.); 3 School of Vehicles and Intelligent Transportation, Fuyao University of Science and Technology, Fuzhou 350300, China

**Keywords:** polyurethane foam, visco-hyperelastic model, finite element method, parameter identification, stress decomposition

## Abstract

Polyurethane foam is widely used as a primary filling material in car seats. While it provides good damping and energy absorption, the mechanical properties are complex but play a vital role in vibration attenuation and vehicle ride comfort. This study proposes a comprehensive experimental and analytical method to characterize the visco-hyperelastic properties of seat-grade polyurethane foam. Quasi-static and dynamic compression tests were conducted on foam blocks to obtain load–deflection curves and dynamic stiffness. A visco-hyperelastic material model was developed, where the hyperelastic response was derived via the hereditary integral and difference-stress method, and viscoelastic behavior was captured using a Prony series fitted to dynamic stiffness data. The model was validated using finite element simulations, showing good agreement with experimental results in both static and dynamic conditions. The proposed method enables accurate characterization of the visco-hyperelastic material properties of seat-grade polyurethane foam.

## 1. Introduction

The passenger car stands as one of the most remarkable inventions in human history, revolutionizing human life by providing convenience and efficiency. In this context, seat design plays a vital role as it serves as the primary interface between occupants and the vehicle structure. Undoubtedly, ensuring comfort has a high rank among the most crucial aspects of seat design. To comprehensively address this objective, the study of vehicle seat comfort can be approached from static and dynamic perspectives, depending on whether the seat is subjected to vibrations or not.

Since the early 1960s, flexible-formed polyurethane foam has emerged as the preferred material for automotive seat applications [1]. The success of this material can be attributed, in part, to its remarkable ability to be tailored to meet diverse performance objectives required by vehicles worldwide. Particularly, its vibration-damping characteristics can be easily customized, allowing manufacturers to optimize comfort levels for occupants. In light of this, understanding the physical characteristics of polyurethane foam and their relationship to seat comfort plays an important role in seat design.

The stress–strain behavior of polyurethane foams exhibits nonlinear hyperelastic characteristics, which can be described by various hyperelastic models [2,3]. These models available in the literature have been developed primarily by fitting experimental data of hyperelastic materials, typically by evaluating the strain energy density derived from the Green–Lagrange tensor or Euler–Almansi tensor. In addition to hyperelasticity, polyurethane foams also exhibit viscoelastic behavior, which significantly influences their performance [4,5]. A material is considered memory viscoelastic if its current response depends not only on its current loading state but also on its previous state [6]. Yang and Shim proposed a visco-hyperelastic model to capture the large three-dimensional compression behavior of foams under strain rates [7]. Ju et al. developed a visco-hyperelastic constitutive model to characterize the quasi-static behavior of polyurethane foam under large deformations, utilizing the difference-stress method to identify the viscoelastic behavior [8]. 

Describing the characteristics of polyurethane foam using the finite element method poses significant challenges. Firstly, elastomeric behavior is inherently complex, and secondly, the material undergoes large deformations leading to geometric nonlinearity. Numerous studies have conducted numerical simulations based on theoretical constitutive models to determine the material properties. Liang and Chandrashekhara developed a neural network-based strain energy function for compressible elastomeric foam and implemented it in the ABAQUS finite element code [9]. Silber et al. assessed the suitability of a strain energy function proposed by Hill and Storakers for finite hyperelasticity in describing the elastic properties of soft foams [10]. Mircheski et al. created a virtual solid model of the body and driver’s seat and performed virtual testing of the sitting process in ABAQUS [11]. Kim et al. calibrated hyperelastic and hyperfoam constitutive models for rigid polyurethane foam, capturing the characteristics of both soft foam and rubber [12]. Grujicic et al. developed detailed finite element models of a car seat and a seated human to investigate their interactions and resulting seating comfort [13]. Oh et al. and Kim et al. incorporated normalized stress relaxation moduli into the static stress–strain curves to predict time-dependent deformation [14,15]. However, they considered viscoelasticity only during relaxation testing, not in static compression tests. Fazekas and Goda proposed numerical stress solutions to calibrate hyper-viscoelastic material models for polymer foams, allowing the separation of time-independent hyperelastic and time-dependent viscoelastic model parameters by mitigating errors [16].

Previous studies have commonly utilized the hyperfoam constitutive model in finite element analyses of low-density foam, with the model parameters determined through curve fitting based on experimental data. However, the hyperfoam model is essentially a hyperelastic model and cannot capture the viscoelastic properties of polyurethane foam. To address this limitation, some researchers attempted to incorporate viscoelasticity directly into hyperelastic models, often by applying stress relaxation functions or simplified assumptions. In these approaches, it is typically assumed that the stress–strain response is time-independent, which may neglect the effects of viscous dissipation observed during loading and unloading in compression tests. Moreover, many of these models treat the total stress as a single outcome of both elastic and viscous effects, without explicitly decomposing the stress components. As a result, they may fail to accurately represent the coupled visco-hyperelastic behavior of polyurethane foam under varying loading conditions.

In this study, a method that independently identifies the hyperelastic and viscoelastic components based on quasi-static and dynamic test data was introduced, enabling accurate parameter determination and numerical implementation. The hyperelastic and viscoelastic components of stress were distinguished and identified independently based on the results of the above-mentioned tests. The results of the numerical calculations demonstrated excellent agreement with the experimental results.

## 2. Experimental Study

Quasi-static and dynamic compression tests are commonly performed on low-density polyurethane foam to investigate its mechanical behavior. In this study, we conducted these two tests to acquire the necessary data for modeling and analysis, which also offer valuable insights into the material’s structural performance under varying loading conditions.

### 2.1. Materials

A polyurethane foam sample provided by a seat supplier (Changchun Hongda Sponge Manufacturing Co., Ltd., Changchun, China) was selected for the quasi-static and dynamic tests. The foam sample had a density of 65 kg/m^3^ and a hardness of 150 N, which was determined using the low indentation hardness index as per method D defined in ISO 2439-2008. Table 1 is a summary of the material properties for the polyurethane foam sample.

### 2.2. Quasi-Static Compression Test

**Figure 1:**
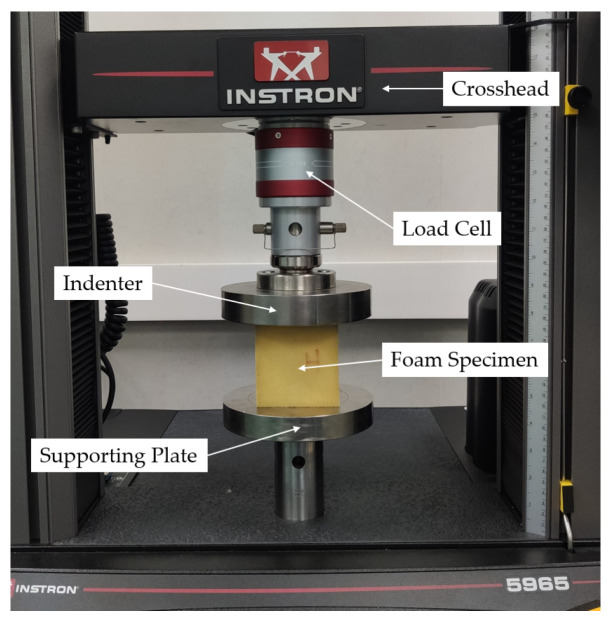
The apparatus used in the quasi-static test.

The quasi-static tests were conducted using an “INSTRON 5965” (Instron, Norwood, MA, USA) universal material testing system equipped with Bluehill Universal Software (v 4.28), with a force measurement accuracy of ±0.5% and a 2.5 kHz data acquisition rate, which fulfilled the requirements of the quasi-static test. This instrument featured a vertically moving actuator, and the indenter was securely bolted to the crosshead of the actuator to cover the upper surface of the specimen. The experimental setup for the quasi-static test is illustrated in Figure 1.

In this study, Method E in ISO 2439-2008 was utilized as a reference for the test procedure. The test specimen was positioned on the supporting plate of the apparatus, and an indentation was made on the specimen. To initiate the test, the specimen underwent a preloading phase at a rate of 120 mm/min, resulting in a 60 mm deformation. The load was released at the same rate until the top of the specimen was fully cleared. Subsequently, the specimen was allowed to rest for a period of 5 min. To commence the compression test, the indenter was lowered at a rate of 30 mm/min until a contact force of 4.5 ± 0.5 N was achieved. At this point, the displacement and force indicators were zeroed, establishing the starting point of the compression test. The specimen was then compressed at a rate of 120 mm/min to 75% of its thickness, while the force–displacement data was continuously recorded. Following the completion of the compression cycle, there was a controlled interval of 2 s before the decompression cycle commenced. The average value of the 6 tests was taken as the final result for the sample.

**Figure 2:**
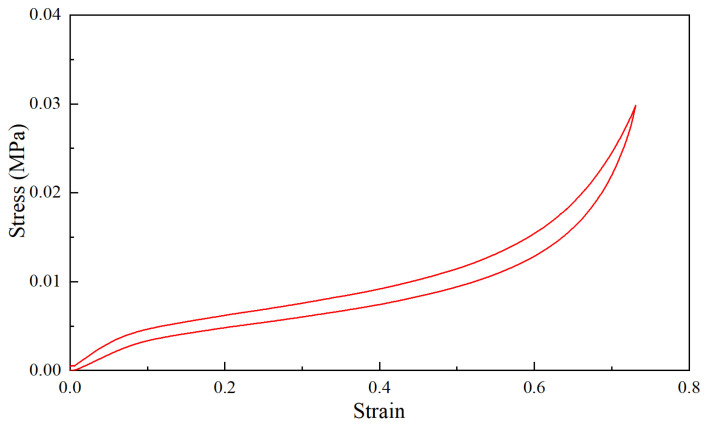
The stress–strain curve of the quasi-static compression test of the sample.

Initially, the obtained force–displacement test results were transformed into nominal stress–strain test results. The nominal strain was calculated as the change in thickness per unit of the original thickness, while the nominal stress was determined as the force per unit of the original cross-sectional area. The resulting stress–strain curve of the polyurethane foam sample is depicted in Figure 2. As shown in the figure, the stress–strain curve exhibited pronounced nonlinear characteristics. Notably, the loading and unloading paths did not overlap, indicating the presence of viscoelastic properties. 

### 2.3. Dynamic Compression Test

**Figure 3:**
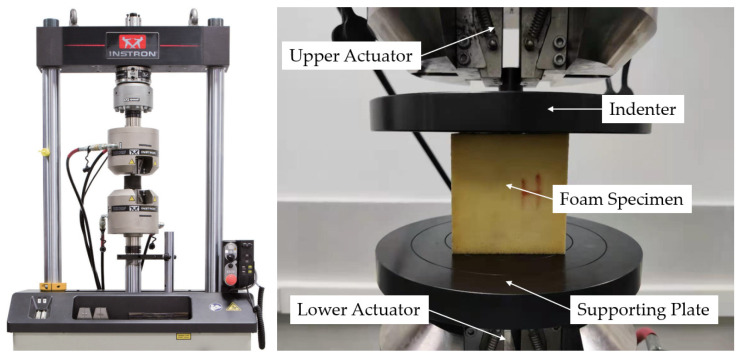
The apparatus used in the dynamic test.

The dynamic test was conducted using the “INSTRON 8801” (Instron, Norwood, MA, USA) servohydraulic fatigue testing system with a force measurement accuracy of ±0.5%, equipped with two inline and vertically moving actuators. To begin the dynamic test, the supporting plate was mounted on the lower actuator and subsequently locked in place. Next, the indenter was securely bolted to the crosshead of the upper actuator. The experimental setup for the dynamic test is depicted in Figure 3.

The dynamic test, described in the “ASTM D3574-2017” standard for constant deflection pounding, served as the reference for this study. The installation and preload procedures were identical to those described in Section 2.2. Subsequently, the specimen was indented at a rate of 30 mm/min to 25% of its thickness. Afterward, the lower actuators produced sinusoidal vibration excitations ranging from 1 to 20 Hz, with frequency intervals of 1 Hz, and at least 20 cycles were executed at each frequency. The amplitude of the vibration excitations at each frequency was set to a peak-to-peak value of 0.25 m/s^2^.

**Figure 4:**
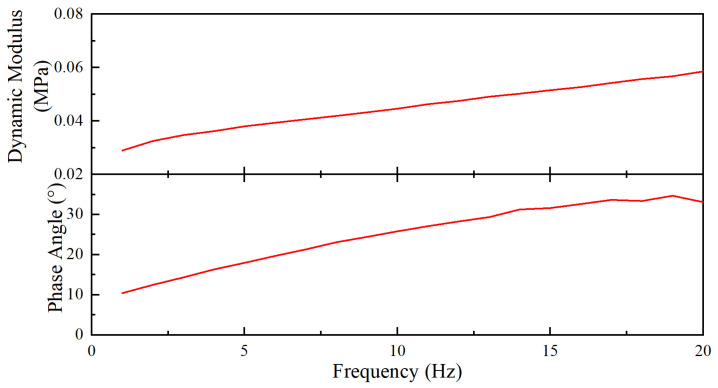
The dynamic modulus and phase angle of the sample.

The force–displacement test results were likewise transformed into nominal stress–strain data. In the context of dynamic testing, the dynamic complex modulus represents the transfer function between the nominal stress and nominal strain. This complex modulus is characterized by three components: the dynamic modulus, which represents its amplitude; the storage modulus, which corresponds to the real part of the complex modulus; and the loss modulus, which represents its imaginary part. The ratio of the loss modulus to the storage modulus is known as the loss factor, and it can be expressed as follows:

(1) $$\tan\delta =\frac{E^{\prime \prime }}{E^{\prime }}$$

where $E^{\prime \prime }$ is the loss modulus, $E^{\prime }$ is the storage modulus, and the angle δ is indicative of the prominence of the viscous effect. The test results are illustrated in Figure 4.

## 3. Parameter Identification of the Visco-Hyperelastic Model of the Foam

This section presents the parameter determination of the visco-hyperelastic model of low-density foam. To achieve this, the material model of the polyurethane foam was accurately defined, and the parameters of the model were determined. To validate the material model, the static and dynamic tests were simulated by finite element analysis. By comparing the calculated results with the corresponding test results, the accuracy of the proposed model was verified.

**Figure 5:**
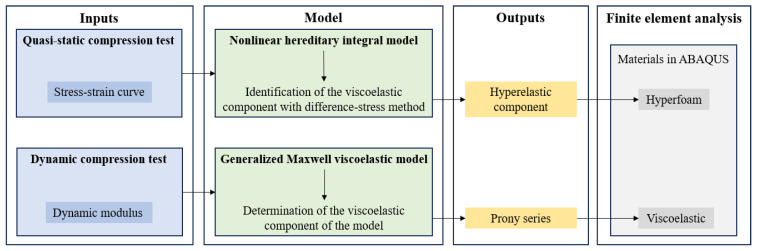
Flow chart of the modeling process.

To predict the mechanical behavior of the foam accurately, a visco-hyperelastic model comprising both nonlinear hyperelastic and viscoelastic components was proposed. “Hyperfoam” and “Viscoelastic” constitutive models were defined in finite element analysis. Specifically, the stress–strain curve obtained from the quasi-static compression test served as the input for identifying the hyperelastic component, while the dynamic modulus data from the dynamic compression test were used to characterize the viscoelastic behavior. Uniaxial compression test data were required for identifying the model parameters when the “Hyperfoam” model was defined. However, given the assumptions in this study, the stress during quasi-static compression tests encompassed both hyperelastic and viscoelastic components. To cope with this challenge, the hereditary integral viscoelastic model and the difference-stress method were employed to decompose the uniaxial compression stress. The resulting hyperelastic part was utilized as input data for the “Hyperfoam” model. Meanwhile, the Prony series was determined through parameter fitting of the dynamic test results to characterize the viscoelastic behavior for the “Viscoelastic” constitutive model. The entire modeling process, detailing the decomposition and fitting steps, is illustrated in Figure 5.

### 3.1. Hyperelastic Model

A modified form of the Hill strain energy potential for constitutive modeling of elastomeric foam materials named “Hyperfoam” in ABAQUS was adopted:

(2) $$U=\sum_{i=1}^{N}\frac{2\mu_{i}}{\alpha_{i}^{2}}\left[{\hat{\lambda }}_{1}^{\alpha_{i}}+{\hat{\lambda }}_{2}^{\alpha_{i}}+{\hat{\lambda }}_{3}^{\alpha_{i}}-3+\frac{1}{\beta_{i}}\left({\left(J^{el}\right)}^{-\alpha_{i}\beta_{i}}-1\right)\right]$$

where $\mu_{i}$, $\alpha_{i}$, and $\beta_{i}$ are temperature-dependent material constants to be determined by curve fitting the experimental stress–strain data; *N* is the curve fitting order.

(3) $${\hat{\lambda }}_{k}={\left(J^{th}\right)}^{-\frac{1}{3}}\lambda_{k}\left(k=1,2,3\right)\;\;{\hat{\lambda }}_{1}{\hat{\lambda }}_{2}{\hat{\lambda }}_{3}=J^{el}$$

Here, $\lambda_{k}$ denotes the principal stretches. The elastic volume ratio, $J^{el}$, relates the total volume ratio *J* and the thermal volume ratio $J^{th}$:

(4) $$J^{el}=\frac{J}{J^{th}}$$

$J^{th}$ is given by

(5) $$J^{th}={\left(1+\varepsilon^{th}\right)}^{3}$$

where $\varepsilon^{th}$ is the linear thermal expansion strain, which is obtained from the temperature and the isotropic thermal expansion coefficient.

When the thermal effect is not considered, which is the case in this paper, $J^{el}=J$ and ${\hat{\lambda }}_{k}=\lambda_{k}$.

It is worth noting that this part only introduced the hyperelastic constitutive model that was utilized in finite element analysis. The hyperfoam constitutive model was not employed for parameter fitting during the determination of the hyperelastic component of the stress from the quasi-static test.

### 3.2. Viscoelastic Model

Two viscoelastic models are introduced in this paper. To distinguish the quasi-static stress, the hereditary integral model, in conjunction with the difference-stress method, was employed. On the other hand, for determining the Prony series with the dynamic compression test, the generalized Maxwell viscoelastic model was utilized.

The viscoelastic stress in this study was characterized using a hereditary integral model, which has been extensively utilized in previous research [17,18,19]. By employing the same loading and unloading parameters, this model effectively separated the stress during the quasi-static compression test through an analytical approach:

(6) $$\sigma_{v}\left(t\right)=\left\{\begin{array}{cc} \int_{0}^{t}\sum_{i=1}^{2}\alpha_{i}e^{-\beta_{i}\left(t-\tau \right)}\varepsilon \left(\tau \right)d\tau & 0\leq t\leq \frac{T}{2}\\ \int_{0}^{\frac{T}{2}}\sum_{i=1}^{2}\alpha_{i}e^{-\beta_{i}\left(t-\tau \right)}\varepsilon \left(\tau \right)d\tau +\int_{\frac{T}{2}}^{t}\sum_{i=1}^{2}\alpha_{i}e^{-\beta_{i}\left(t-\tau \right)}\varepsilon \left(\tau \right)d\tau & \frac{T}{2}\leq t\leq T \end{array}\right.$$

ε is the strain in the test, and $\sigma_{v}$ is the predicted viscoelastic stress response of the test. $\alpha_{i}$ is the *i*^th^ complex number that represents the viscoelastic residue. $\beta_{i}$ is the *i*^th^ complex number that represents the viscoelastic mode.

**Figure 6:**
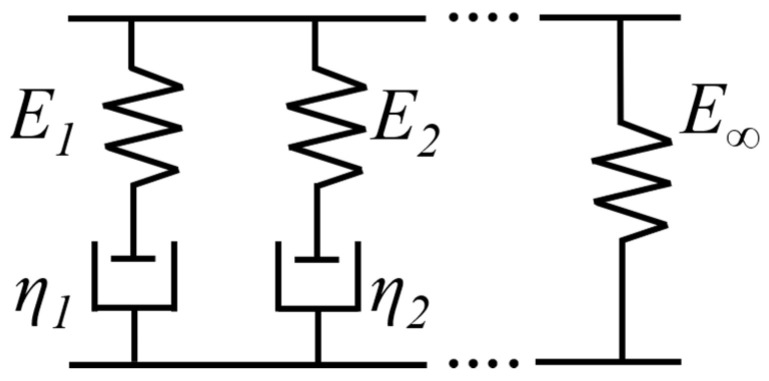
A schematic of the generalized Maxwell model.

To acquire the constitutive model parameters essential for the finite element analysis, the generalized Maxwell model was chosen and represented using the Dirichlet–Prony series. These parameters were determined through dynamic compression tests. The generalized Maxwell model comprises *n* + 1 constituent elements in parallel, which consist of *n* Maxwell models along with an isolated spring, as depicted in Figure 6.

### 3.3. Determining the Hyperelastic Component of the Model

During the quasi-static test, the hyperelastic model effectively captured the material’s nonlinear elastic behavior, while the hereditary integral model accurately depicted the nonlinear viscoelastic response. By integrating the hyperelastic and hereditary integral models, this study established a visco-hyperelastic framework capable of describing the nonlinear mechanical response of polyurethane foam. The stress response of the visco-hyperelastic model could be decomposed into the hyperelastic part and the viscoelastic part:

(7) $$\sigma =\sigma_{e}+\sigma_{v}$$

where σ is the total stress response of the process, and $\sigma_{e}$ and $\sigma_{v}$ are the hyperelastic stress and viscoelastic stress, respectively.

In this study, the difference-stress method was employed to ascertain the viscoelastic parameters. The essence of the difference-stress method lies in leveraging the symmetry of elastic stress during both loading and unloading processes. As a result, the analytical expression for stress difference between loading and unloading solely encompassed the viscoelastic parameters:

(8) $$\delta \sigma =\sigma_{e}\left(t_{1}\right)-\sigma_{e}\left(t_{2}\right)+\sigma_{v}\left(t_{1}\right)-\sigma_{v}\left(t_{2}\right)=\sigma_{v}\left(t_{1}\right)-\sigma_{v}\left(t_{2}\right)$$

where $t_{1}\in \left[0,\frac{T}{2}\right]$ and $t_{2}\in \left[\frac{T}{2},T\right]$ are the loading time and unloading time corresponding to the same strain level. During the quasi-static uniaxial compression test, the strain rate remained constant and consistent between the loading and unloading stages. The relationship between stress and time during these stages was described as follows:

(9) $$\varepsilon \left(\tau \right)=\left\{\begin{array}{cc} \frac{2\varepsilon_{\max}}{T}\tau & 0\leq \tau \leq \frac{T}{2}\\ -\frac{2\varepsilon_{\max}}{T}\tau +2\varepsilon_{\max} & \frac{T}{2}\leq \tau \leq T \end{array}\right.$$

where $\varepsilon_{\max}$ is the maximum strain. The following relationship between $t_{1}$ and $t_{2}$ was not difficult to deduce:

(10) $$t_{1}+t_{2}=T$$

Stress difference could be explicitly expressed by $t_{1}$, and viscoelastic parameters could be expressed by combining Equations (6), (8), and (10). The viscoelastic parameters were ascertained through a parameter optimization method. Utilizing the nonlinear least-square solver and the trust-region reflective algorithm within the MATLAB optimization tool, the error between the model results and the test data was minimized. To quantify the error and evaluate the fitting accuracy, the root mean square error function was employed:

(11) $$RMSE=\sqrt{\frac{1}{n}\sum_{i=1}^{n}{\left[\sigma_{\mathrm{mod}}\left(t_{1}^{\left(i\right)}\right)-\sigma_{\exp}\left(t_{1}^{\left(i\right)}\right)\right]}^{2}}$$

where $\sigma_{\mathrm{mod}}$ is the modeled stress and $\sigma_{\exp}$ is the experimental stress. In this study, the computation of these two stresses was conducted at intervals of 1 s. $t_{1}^{\left(i\right)}$ represents the selected discrete time point. The maximum index value *n* of the loading time *i* was set to 150, corresponding to the completion of loading at 150 s.

**Figure 7:**
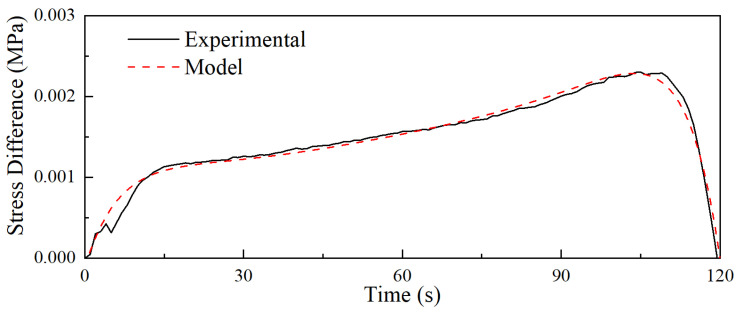
The comparison of stress differences between the model and the test.

The viscoelastic parameters were ultimately obtained and are presented in Table 2. To assess the model’s accuracy, a comparison of stress differences between the model and the test data is depicted in Figure 7.

**Figure 8:**
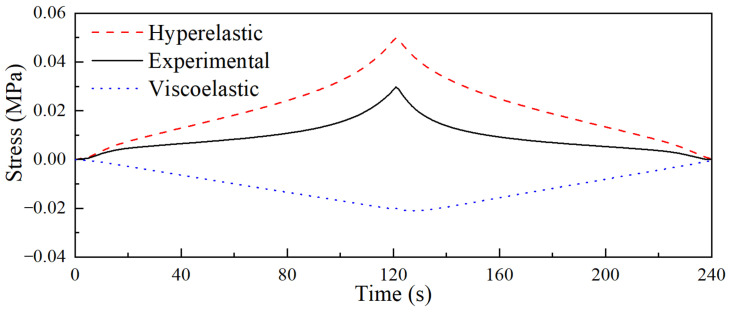
The decomposed hyperelastic and viscoelastic stress.

The viscoelastic part of the quasi-static test, in both loading and unloading stages, was calculated based on the obtained viscoelastic parameters. Meanwhile, the hyperelastic part was computed using Equations (6) and (7). The resultant decomposed hyperelastic and viscoelastic stresses are illustrated in Figure 8.

### 3.4. Determining the Parameters of the Viscoelastic Model

In this section, the parameters of the generalized Maxwell viscoelastic model were determined through the dynamic modulus test. The relaxation modulus of the generalized Maxwell model is represented as follows:

(12) $$E\left(t-\tau \right)=E_{\infty }+\sum_{r=1}^{n}E_{r}e^{-\frac{t-\tau }{T_{r}}}$$

where $E_{\infty }$ represents the equilibrium modulus to maintain the long-term elasticity of the model. $T_{r}=\eta_{r}/E_{r}$ represents relaxation time. $E_{r}$ is the elastic modulus, and $\eta_{r}$ is the viscosity coefficient.

When a viscoelastic material is subjected to an oscillating strain, the controlled strain load can be expressed as follows:

(13) $$\varepsilon \left(t\right)=\varepsilon_{0}e^{i\omega t}=\varepsilon_{0}\left(\cos\omega t+i\sin\omega t\right)$$

where $\varepsilon_{0}$ is the strain amplitude, and ω is the angular velocity. The dynamic Prony series expression of the generalized Maxwell model can be obtained:

(14) $$E^{*}\left(i\omega \right)=E_{\infty }+\sum_{r=1}^{n}\frac{i\omega E_{r}\eta_{r}}{i\omega \eta_{r}+E_{r}}$$

(15) $$E^{\prime }\left(\omega \right)=E_{\infty }+\sum_{r=1}^{n}\frac{\omega^{2}E_{r}\eta_{r}^{2}}{\omega^{2}\eta_{r}^{2}+E_{r}^{2}}=E_{\infty }+\sum_{r=1}^{n}\frac{\omega^{2}T_{r}^{2}E_{r}}{\omega^{2}T_{r}^{2}+1}$$

(16) $$E^{\prime \prime }\left(\omega \right)=\sum_{r=1}^{n}\frac{\omega E_{r}^{2}\eta_{r}}{\omega^{2}\eta_{r}^{2}+E_{r}^{2}}=\sum_{r=1}^{n}\frac{\omega T_{r}E_{r}}{\omega^{2}T_{r}^{2}+1}$$

$E^{*}\left(i\omega \right)$ represents the complex modulus, and $E^{\prime }\left(\omega \right)$ and $E^{\prime \prime }\left(\omega \right)$ denote the real and imaginary parts of the complex modulus, respectively. The phase angle δ represents the viscous effect of the material.

For the discrete dynamic modulus frequency domain data, the relaxation time of each Maxwell sub-model was manually preset in advance, and parameters like equilibrium modulus and elastic modulus were determined through fitting the test data. In this study, the relaxation times of each artificially configured Maxwell sub-model were set as 0.01s, 0.1s, 1s, 10s, and 100s, respectively.

The Prony series parameters, obtained from the storage and loss moduli cyclic test data at M frequencies, were determined by minimizing the error function $\chi^{2}$ to achieve the most accurate fit between the model predictions and the test data:

(17) $$\chi^{2}=\sum_{i=1}^{M}\frac{1}{E_{\infty }^{2}}\left[{\left(E^{\prime }-{\bar{E}}^{\prime }\right)}_{i}^{2}+{\left(E^{\prime \prime }-{\bar{E}}^{\prime \prime }\right)}_{i}^{2}\right]$$

where ${\bar{E}}^{\prime }$ and ${\bar{E}}^{\prime \prime }$ are the test data. In this paper, *M* is 20. Prony series were obtained as shown in Table 3.

## 4. Model Validation of the Foam by Finite Element Analysis 

To validate the constructed material model, the test processes on the foam on a larger scale (400 × 400 × 80 mm), but with the same material properties as in Section 2, were simulated using the finite element method.

### 4.1. Finite Element Model

**Figure 9:**
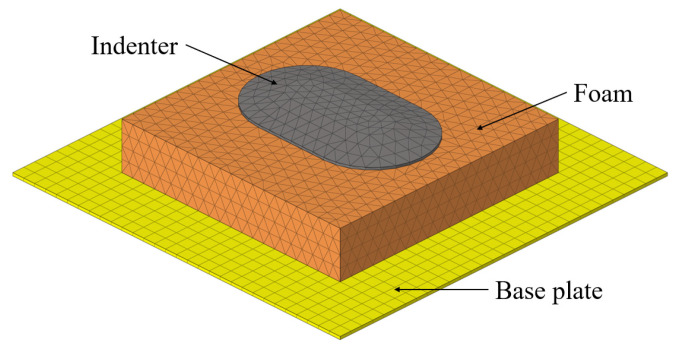
The finite element model is composed of the base plate, the polyurethane foam, and the indenter.

As depicted in Figure 9, the finite element model comprised three primary components. A SIT-BAR commonly employed in seat comfort research was utilized as the indenter. In the previous section, the property definition of polyurethane foam in ABAQUS was briefly introduced. For polyurethane foam, two models were defined: the “Hyperfoam” hyperelastic model and the “Viscoelastic” model. The stress–strain curve obtained from the quasi-static uniaxial compression test (as discussed in Section 3.3) was utilized in the “Hyperfoam” model, with the strain energy potential curve fitting order set to 3. In the “Viscoelastic” model, the Prony series corresponding to each preconfigured relaxation time (from Section 3.4) was employed. To consider the structural damping of polyurethane foam, Rayleigh damping was added to the model. *α* = 180 and *β* = 1 × 10^−4^ were obtained by parameter optimization.

### 4.2. Finite Element Analysis

In the quasi-static analysis, the “Visco” step was employed to study problems involving time-dependent material response, with a total time of 240 s. Contact was defined to obtain the contact force between the indenter and the polyurethane foam. All degrees of freedom of nodes on the base plate and the indenter (excluding the Z-direction) were constrained. Following the test process, the indenter was uniformly pressed down 60 mm at a constant speed during the initial 120 s and then moved up at the same speed.

The initial contact and constraint setup for the dynamic process analysis remained consistent with the quasi-static analysis. The dynamic analysis was divided into two steps. In the first step, “Visco”, the indenter descended 20 mm at a constant speed over 40 s and then was fixed in position. The second step involved “steady-static dynamics,” where the constraint in the Z-direction of the bottom plate was removed. Displacements corresponding to an amplitude of 0.25 m/s^2^ were applied in the frequency range of 1–20 Hz, with a frequency interval of 1 Hz.

### 4.3. Results

**Figure 10:**
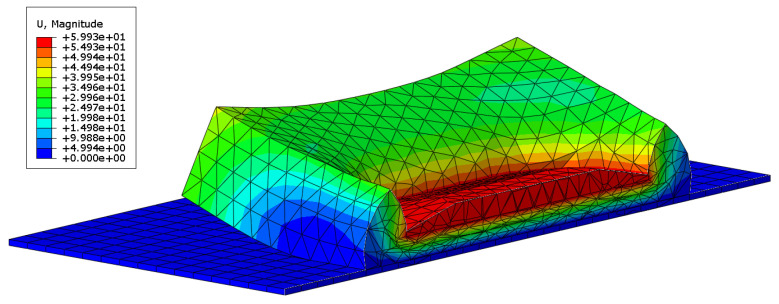
The deformation at the indentation of 60 mm by quasi-static analysis.

**Figure 11:**
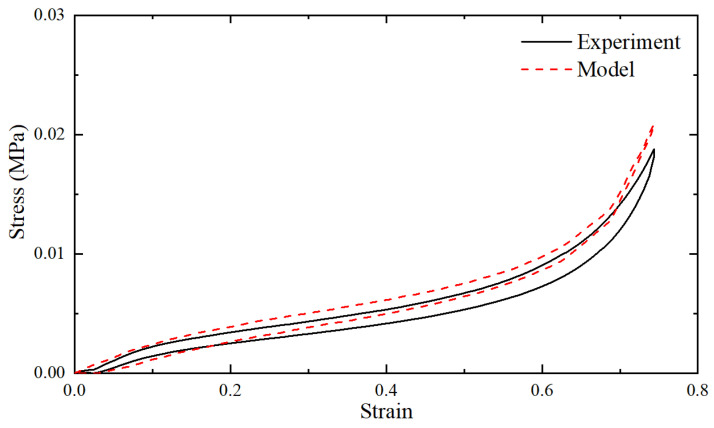
The comparison between the reaction forces of the indenter obtained by quasi-static analysis and the test.

**Figure 12:**
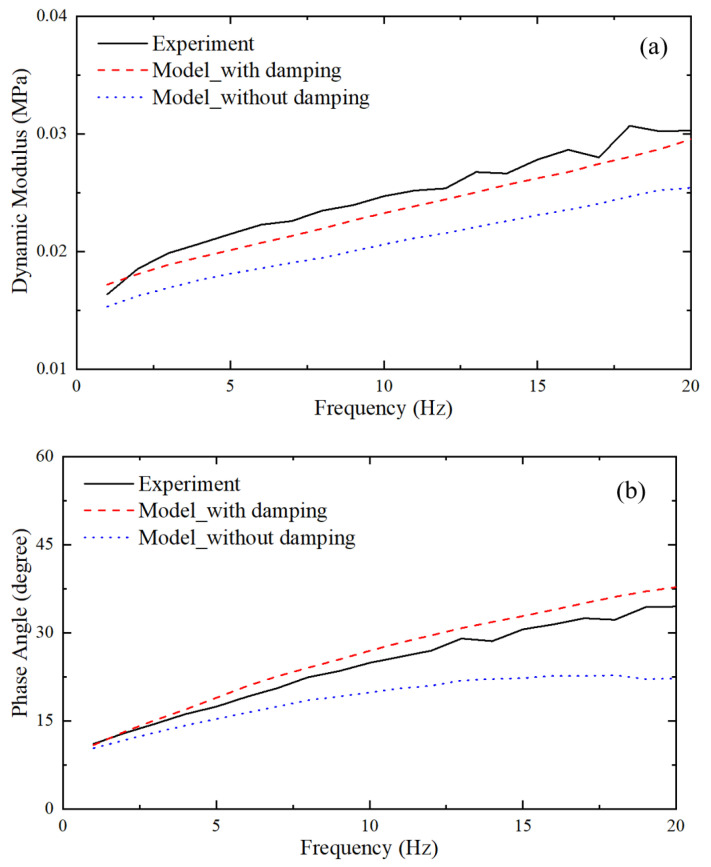
The comparison between the (**a**) dynamic modulus, (**b**) phase angle obtained by dynamic analysis, and the test results.

Figure 10 presents the deformation at the 60 mm indentation obtained through quasi-static analysis. Figure 11 shows the comparison between the contact pressure of the indenter obtained from quasi-static analysis and the nominal stress from the test. Figure 12 displays the comparison of dynamic modulus and phase angle obtained through dynamic analysis with the corresponding test results.

## 5. Discussion

**Figure 13:**
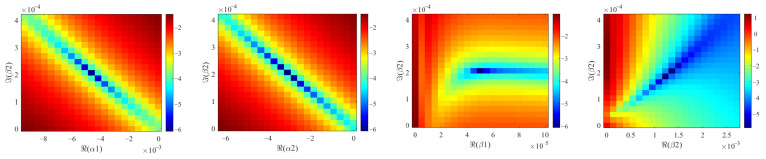

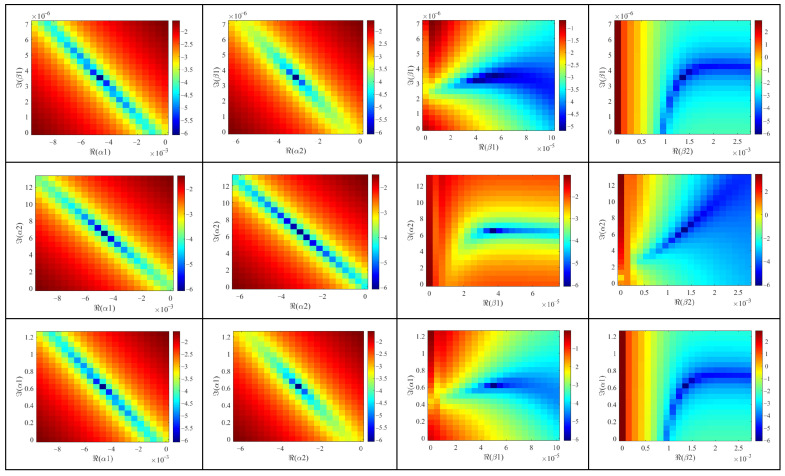
The error surfaces of the viscoelastic parameters in the real–imaginary planes.

To obtain the hyperelastic part using the hereditary integral model of viscoelastic stress, we employed the trust-region reflective algorithm with tight stopping criteria, which effectively handled the rapidly changing objective function. The error is shown in Figure 13. The hereditary integral model had two pairs of complex viscoelastic parameters, totaling eight parameters that significantly influenced the model’s accuracy. 

As depicted in Figure 13, the horizontal and vertical coordinates represented the real and imaginary parts of each parameter, respectively, while the color bar depicted the logarithmic form of the error RMSE in Equation (11). The parameters were varied from 0 to double the determined values, leading to the minimum error consistently emerging at the center of each error surface.

It is evident that surfaces with the same subscript for imaginary parts (*α*_1_ and *β*_1_; *α*_2_ and *β*_2_) exhibited similar patterns. Therefore, we will focus on discussing surfaces with imaginary parts of *α*_1_ and *α*_2_ below. Regarding surfaces with real parts of *α*_1_ and *α*_2_, smaller errors were observed along the diagonal, and the degree of dependence on parameter changes varied for each parameter. As for surfaces with real parts of *β*_1_ and *β*_2_, in conjunction with the aforementioned imaginary parts of *α*_1_ and *α*_2_, each surface displayed a distinct pattern but exhibited similar characteristics. When the real parts were smaller than the determined values, the error was significantly influenced by the real parts, whereas when the real parts were larger than the determined values, the error was predominantly influenced by the imaginary parts.

Secondly, the analysis result closely matched the experimental result, clearly demonstrating the hysteresis effect. The observed error may mainly arise from the following factors:

1. Polyurethane foam exhibits a high sensitivity to strain rate, primarily caused by the expulsion of air from the foam cells during the deformation of open-cell foams. This aspect was not taken into account in the current study.

2. The error may be attributed to the determination of the viscoelastic Prony series based on the dynamic compression test results. The conditions of the dynamic tests, such as preload and vibration amplitude, could potentially influence the final values of the Prony series. Further investigations and refinement of the test conditions may lead to improved accuracy in the model predictions.

Thirdly, it is evident from Figure 12 that incorporating additional damping factors, apart from viscoelastic damping, yielded more accurate results. These additional damping factors are primarily attributed to air passing through the foam at higher frequencies during dynamic tests. Many other researchers have also considered these damping factors in their studies [20,21].

The proposed model demonstrated satisfactory accuracy in the realm of seat comfort research. Compared with existing visco-hyperelastic models proposed in previous studies, the present work emphasizes a more explicit decomposition of hyperelastic and viscoelastic components through the combination of the hereditary integral model and the difference-stress method. While the model in [7] effectively captures large-strain behavior under dynamic loading, and the model in [8] focuses on quasi-static conditions using the difference-stress method, our approach integrates both quasi-static and dynamic tests, enabling independent identification of each component and facilitating direct implementation in commercial finite element software ABAQUS 2022. This enhances the model’s applicability to practical seat comfort analysis. Moreover, the tests employed in finite element analysis are widely used in seat comfort assessments. The findings of this study provided an accurate representation of the nonlinear and time-dependent behavior of polyurethane foam, which is essential for improving seat comfort prediction. The proposed model may assist in optimizing foam geometry, layering strategies, and material selection in automotive seat design.

For future investigations, additional test modes, such as the vibration transmissibility test, tensile test, and shear test, could be considered to further validate the model. Furthermore, the current study focuses on a specific type of polyurethane foam commonly used in car seat applications. While the proposed visco-hyperelastic model effectively captures its mechanical behavior, the applicability of the model to other foam types—such as those with lower viscosity, PUR Polyester, PUR Polyether, or multi-component systems—remains to be investigated. Future work will explore the model’s adaptability and parameter identification procedures for these alternative materials to evaluate its broader applicability.

## 6. Conclusions

In this paper, a novel parameter identification method was proposed to accurately characterize the mechanical properties of polyurethane foam for further finite element analysis, using quasi-static and dynamic stiffness tests commonly conducted in the seat comfort analysis. By employing the visco-hyperelastic model, the hyperelastic component of the compression stress in the quasi-static test was determined through the combination of the hereditary integral model and the difference-stress method. The viscoelastic Prony series was derived from the dynamic test using the generalized Maxwell model. The proposed constitutive model, “Hyperfoam,” and the viscoelastic Prony series were implemented in ABAQUS for numerical computations. The results obtained from the quasi-static compression and dynamic compression analyses demonstrated satisfactory accuracy, validating the effectiveness of the proposed method in accurately capturing the behavior of polyurethane foam.

## Figures and Tables

**Table 1:** Material properties for the polyurethane foam sample.

Foam Type	Flexible Polyurethane Foam
Isocyanate	Toluene diisocyanate
Polyol	Polyether
Expansion gas	CO_2_
Cell type	Open
Dimension (l × w × h)	80 mm × 80 mm × 80 mm
Density	65 kg/m^3^
Hardness	150 N

**Table 2:** The determined viscoelastic parameters.

Re (*α*_1_)	Im (*α*_1_)	Re (*β*_1_)	Im (*β*_1_)	Re (*α*_2_)	Im (*α*_2_)	Re (*β*_2_)	Im (*β*_2_)
−4.85 × 10^−3^	0.662	5.25 × 10^−5^	3.45 × 10^−6^	−3.01	6.25	1.32 × 10^−3^	2.01 × 10^−4^

**Table 3:** The determined Prony series.

Relaxation Time	*T*_1_ (s)	*T*_2_ (s)	*T*_3_ (s)	*T*_4_ (s)	*T*_5_ (s)	-
	0.01	0.1	1	10	100	-
Prony Series	*E*_1_ (kPa)	*E*_2_ (kPa)	*E*_3_ (kPa)	*E*_4_ (kPa)	*E*_5_ (kPa)	*E*_∞_ (kPa)
	23.9	19.7	8.99	12.4	8.14	3.05

## Data Availability

The original contributions presented in this study are included in the article. Further inquiries can be directed to the corresponding authors.
